# Association between quality of care indicators and clinical outcomes in patients undergoing transcatheter aortic valve implantation: insights from SWEDEHEART

**DOI:** 10.1093/ehjqcco/qcaf146

**Published:** 2025-12-03

**Authors:** Konrad Nilsson, Stefan James, Jenny Backes, Matthias Götberg, Henrik Hagström, Gorav Batra

**Affiliations:** Cardiology, Department of Medical Sciences, Uppsala University, Akademiska sjukhuset ing. 40, SE-751 85 Uppsala, Sweden; Uppsala Clinical Research Center, Dag Hammarskjölds väg 38, SE-751 85 Uppsala, Sweden; Nuffield Department of Population Health, University of Oxford, Richard Doll Building, Old Road Campus, Oxford OX3 7LF, UK; Cardiology, Department of Medical Sciences, Uppsala University, Akademiska sjukhuset ing. 40, SE-751 85 Uppsala, Sweden; Uppsala Clinical Research Center, Dag Hammarskjölds väg 38, SE-751 85 Uppsala, Sweden; School of Medical Sciences, Faculty of Medicine and Health, Örebro University, Campus USÖ, SE-701 82 Örebro, Sweden; Department of Cardiothoracic and Vascular Surgery, Örebro University Hospital, SE-703 82 Örebro, Sweden; Cardiology Unit, Department of Clinical Sciences Lund, Lund University, Sölvegatan 19, SE-223 62 Lund, Sweden; Department of Cardiology, Skåne University Hospital, Entrégatan 7, SE-222 42 Lund, Sweden; Department of Public Health and Clinical Medicine, Umeå University, SE-901 87 Umeå, Sweden; Heart Centre, Umeå University Hospital, Daniel Naezéns väg, SE-907 37 Umeå, Sweden; Cardiology, Department of Medical Sciences, Uppsala University, Akademiska sjukhuset ing. 40, SE-751 85 Uppsala, Sweden; Uppsala Clinical Research Center, Dag Hammarskjölds väg 38, SE-751 85 Uppsala, Sweden

**Keywords:** Transcatheter aortic valve implantation, Quality of care, Quality indicators, Cardiovascular outcomes, Implementation, Mortality

## Abstract

**Aims:**

The European Society of Cardiology (ESC) has developed quality indicators (QIs) specifically for evaluating the care and outcomes for patients undergoing transcatheter aortic valve implantation (TAVI). The aim of this study was to evaluate TAVI care in all patients undergoing such procedures in Sweden using the ESC 2023 QIs for TAVI.

**Methods and results:**

We used the Swedish Transcatheter Cardiac Intervention Registry to identify all TAVI procedures performed in Swedish centres (*n* = 8) between 2008 and 2021. 8524 patients (median 82 years, 52.8% male) were included. In total, 24 (88.8%) of the 27 QIs could be captured from the available registry data. The attainment levels were generally high [median 97.8%, interquartile range (IQR) 91.3–100%] with small variations in attainment levels between centres. The greatest variations were observed in the QIs related to patients undergoing TAVI through transfemoral route (median 90.3%, IQR 85.6–96.9%), and in the proportion undergoing transfemoral TAVI without general anaesthesia (median 87.6%, IQR 57.1–94.4%). In total, 80% of the QIs were associated with 1-year all-cause mortality, and 85% with 1-year cardiovascular mortality. The QIs with greatest impact on 1-year mortality were the absence of coronary obstruction (adjusted HR 0.12; 95% CI 0.07–0.22), in-hospital stroke (adjusted HR 0.25; 95% CI 0.18–0.33) and no new dialysis (adjusted HR 0.25; 95% CI 0.15–0.41).

**Conclusion:**

In this study, the majority of the QIs were associated with both all-cause and cardiovascular mortality. Hence, the ESC 2023 QIs for TAVI may serve as a valuable tool for evaluating TAVI care, benchmarking performance, and improving patient outcomes.

## Graphical Abstract

**Graphical Abstract:**
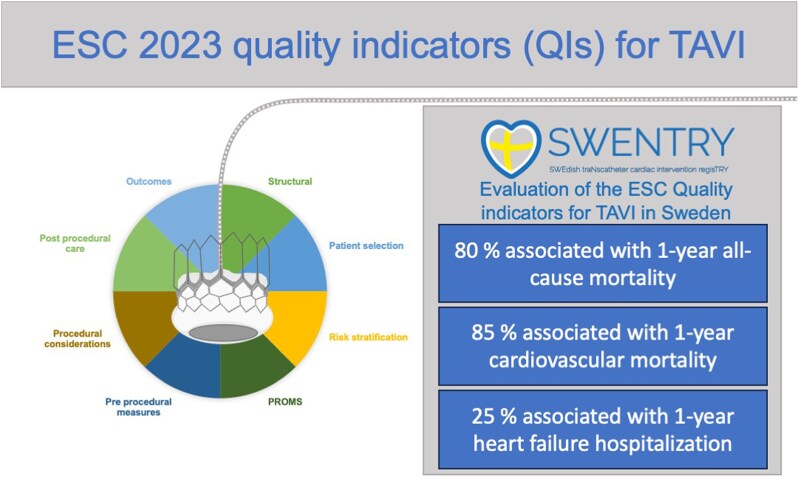

Key Learning Points What is already known:

- Transcatheter aortic valve implantation (TAVI) is an increasingly important treatment option for patients with aortic stenosis.
- Quality indicators (QIs) may have an important role in ensuring high-quality care.
- The European Society of Cardiology (ESC) has developed QIs specifically for evaluating the care and outcomes for patients undergoing TAVI.

What this study adds:

- The ESC 2023 QIs for TAVI can be captured from existing registry data.
- The majority of the QIs were associated with both all-cause and cardiovascular mortality.
- The ESC 2023 QIs for TAVI may serve as a valuable tool for evaluating TAVI care, benchmarking performance, and improving patient outcomes.

## Introduction

Transcatheter Aortic Valve Implantation (TAVI) has become an established procedure for treating severe aortic stenosis, particularly in patients who are considered high or intermediate risk for conventional surgical aortic valve replacement.^1^ Over the past decade, the use of TAVI has rapidly expanded due to its minimally invasive nature, faster recovery times, and comparable outcomes to surgical interventions.^2-6^ As the procedure becomes more widely available, ensuring high-quality care and optimal patient outcomes is increasingly important, and here quality indicators (QIs) might have an important role.^7^

The European Society of Cardiology (ESC) has developed QIs specifically for evaluating the care and outcomes for patients undergoing TAVI.^8^ These indicators are intended to serve as benchmarks for assessing the quality of care and guiding healthcare systems in continuously improving clinical practice. The ESC QIs are categorized into structural, process, and outcome indicators, covering key aspects such as patient selection, procedural safety, long-term follow-up, and complication management.^8^

The QIs have been implemented in the European Unified Registries for Heart Care Evaluation and Randomized Trials (EuroHeart) TAVI registry, thereby enabling measurement and improvement of European TAVI care.^8,9^ To further establish the usefulness of the TAVI QIs, an evaluation of the quality of care against the QIs is warranted. The Swedish Web-system for Enhancement and Development of Evidence-based care in Heart disease Evaluated According to Recommended Therapies (SWEDEHEART)^10^ has national coverage of all Swedish TAVI procedures and was one of several existing registries that was evaluated during the development of the data standards for TAVI by EuroHeart.^9^ As a consequence, the SWEDEHEART registry is suitable for investigating if the proposed QIs are possible to extract using existing registry data, and if they are associated with relevant clinical outcomes, which was the aim of this study.

## Methods

The data underlying this study were collected from several SWEDEHEART sub-registries described below. Data on TAVI procedures were obtained from the Swedish Transcatheter Cardiac Intervention Registry (SWENTRY). SWENTRY holds information on comorbidities, echocardiographic findings and procedural measurements for all patients undergoing TAVI in Sweden. The Swedish Cardiac Surgery Registry, contains detailed information on comorbidities, surgical considerations and short-term outcomes, which was used to identify reinterventions. The Swedish Coronary Angiography and Angioplasty Registry (SCAAR) holds detailed information on coronary angiographies and percutaneous interventions, which was used to identify patients with a recent percutaneous coronary intervention (PCI) prior to TAVI. Using the unique personal identification number available to all Swedish citizens, data were further linked to the National Patient Register^11^ to identify rehospitalizations, and to the National Cause of Death Register^12^ to obtain dates and causes of death. Both these registers are mandatory nationwide registries which collect information on dates and diagnoses based on the International Statistical Classification of Diseases and Related Health Problems—Tenth Revision (ICD-10) system for patient visits to inpatient and outpatient clinics and dates and causes of death, respectively. Cardiovascular death was defined as death with ICD-10 code starting with ‘I’ as main diagnosis. Heart failure hospitalization was defined as a hospitalization in the National Patient Registry with I11, I13, I13.2, I25.5, I42.0, I42.3, I42.5, I42.6, I42.7, I42.8, I42.9, K76.1, R57 or ICD-10 codes starting with I43, I50 or J81 as main diagnosis. The National Board of Health and Welfare in Sweden conducted the linkage of data which were then delivered fully pseudonymized for analysis and reporting.

The patient cohort consisted of all consecutive patients undergoing TAVI due to severe aortic stenosis from 1 January 2008 to 31 December 2021 in Sweden. Index date was defined as procedure date. Age below 18 years was used as an exclusion criterion.

### Quality indicators for TAVI

The ESC 2023 QIs for TAVI^8^ spans eight domains of care for the management of TAVI: (i) Structural framework, (ii) Patient selection, (iii) Risk stratification, (iv) Patient reported outcomes measures (PROM)s (v) Pre-procedural measures, (vi) Procedural considerations, (vii) Post-procedural care and (viii) Outcomes. For each QI, the eligibility criteria (denominator) and the accomplishment criteria (numerator) were derived from the corresponding data variables in the SWENTRY registry. In total, 24 out of 27 QIs could be captured using data from the registries, of these 21 were obtained unaltered from existing variables, while 3 required slight modifications. Frailty was captured, however, unlike what was suggested in the published QIs, it was not based on a validated instrument. For this study, we chose to use the existing SWENTRY variable, although it may have been influenced by the operator’s subjective assessment. The two remaining modifications involved the need for data about recent PCI. However, this information was not available in SWENTRY and instead, data were enriched using information from the SCAAR registry. Attainment of QIs was analysed for all eligible patients and stratified by clinical centre. In addition, stratified analyses were performed by age (above or below median) and by sex.

Three QIs could not be calculated for the entire study period and were only assessed during the years when the necessary variables were available: (i) Number of patients considered for TAVI who had a pre-procedural cardiac-gated computed tomography (CT) scan was included as a variable from 1 January 2016; (ii) Prescribed medications were included from 4 June 2019, leading to that the variables measuring proportion of patients with atrial fibrillation (AF) who were on oral anticoagulation (OAC) monotherapy and number of patients without recent PCI on single anti-platelet therapy (SAPT) were analysed from that date.

The number of patients per centres varied between years. A high-volume centre was defined as a centre with more than 100 procedures during 2021. In the published version of the ESC 2023 QIs for TAVI, dialysis and major bleeding are defined as positive statements whereas the rest of the clinical outcomes are defined as negations, e.g. ‘no cardiac tamponade’. To facilitate the interpretation of the results, we chose to inverse the definitions of dialysis and major bleeding throughout this publication.

### Outcomes

The primary outcome was all-cause mortality at 1 year. Secondary outcomes included cardiovascular mortality and hospitalization for heart failure at 1 year. Outcome data were obtained from the National Cause of Death Register and the National Patient Register. The last day of follow-up was 31 December 2021.

### Statistical analysis

Reporting of this study was performed in accordance with Strengthening the Reporting of Observational studies in Epidemiology (STROBE)^13^ statement. Numbers and percentages were used for reporting categorical data, median and interquartile range was used for continuous data. The eligibility (denominator) and accomplishment among eligible patients (numerator) were calculated for each QI. Differences in attainment of QIs between males and females and age below or above median (82 years) were modelled using logistic regression. Attainment of indicators for all patients at each hospital centre was illustrated using box plots. The association between QIs and outcomes was determined using crude and adjusted Cox regression analysis. Adjustment was made, in accordance with similar publications, for the following clinically relevant variables: age, sex, procedure year, New York Heart Association (NYHA) class, diabetes, previous myocardial infarction, previous cardiac surgery, previous stroke, AF, peripheral vessel disease, chronic pulmonary disease, anaemia and estimated glomerular filtration rate (eGFR). Proportional hazards assumptions were assessed using Schoenfeld residuals. Due to signs of non-proportional hazards, sex was stratified and eGFR was modelled using natural cubic splines with 4 degrees of freedom. Censoring was performed at the end of follow-up, 31 December 2021 or, for cardiovascular mortality, other causes of death. In the Cox proportional hazard regression analysis, imputation of missing data was performed by multiple imputation by chained equations with 5 generated datasets including the same variables as in the adjusted Cox regression with the addition of all-cause mortality as Nelson-Aalen estimator.^14^ No imputation of the QIs was performed. The R package ‘mice’^15^ was used for the analysis. All statistical tests were two-sided with *P*-value <0.05 considered as statistically significant. All analyses were performed using R version 4.3.1.^16^

### Data approval and ethics

The study was approved by the Swedish Ethical Review Authority (application number 2017/455 with amendments).

## Results

A total of 8524 patients who underwent TAVI in Sweden between 1 January 2008 and 31 December 2021 across eight centres were included. The median age was 82 years (IQR 77–86), 52.8% were male, and as illustrated in Supplementary material online, *Figure S1*, the number of procedures increased over time. Baseline characteristics are presented in *Table 1*. The level of missing baseline information was overall low, except for pacemaker status and NT-proBNP level data in patient characteristics, and implantation of new permanent pacemaker and the presence of any major bleeding in patient outcomes. For new permanent pacemaker and major bleedings, the missing data occurred in the first half of the study period. After an update of the variables in 2016 their levels of missingness were low, see Supplementary material online, *Figure S2*. Centre variation, presented as median and IQR, was small. Baseline characteristics stratified by median age (82 years) and sex are reported in Supplementary material online, *Table S1*. Among patients above the median age, female sex was more prevalent, and NT-proBNP levels were higher, whereas diabetes, chronic pulmonary disease and dialysis were less common. Meanwhile, females had lower rates of prior PCI and lower serum creatinine levels. However, overall differences were minor.

**Table 1:** Baseline characteristics and hospital variation in characteristics

Characteristic	Overall	Missing n (%)	Hospital variance Median (IQR)
*N*	8524		
Age median [IQR]	82.0 [77, 86]	0 (0)	81.3 [80.8, 81.5]
Sex male *n* (%)	4501 (52.8)	0 (0)	52.7 [51.9, 54.4]
BMI mean (SD)	26.9 (8.4)	38 (0.4)	26.75 [26.6, 27.2]
Year category *n* (%)			
2007–2012	847 (9.9)		9.45 [7.6, 10.9]
2013–2018	3921 (46.0)		45.4 [44.5, 47.3]
2019–2022	3756 (44.1)		44.4 [43.2, 49.8]
Hypertension *n* (%)	6484 (76.1)	2 (0)	80.45 [74.3, 80.7]
Diabetes *n* (%)	2152 (25.3)	2 (0)	26.3 [25.2, 26.8]
Prior myocardial infarction *n* (%)	360 (4.2)	2 (0)	3.25 [3, 4.1]
Prior PCI *n* (%)	2301 (27.0)	2 (0)	27.35 [24.7, 28.8]
Prior stroke *n* (%)	1041 (12.2)		12.3 [11.7, 12.8]
Atrial fibrillation *n* (%)	3147 (36.9)	0 (0)	36.9 [36.4, 38.2]
Peripheral vessel disease *n* (%)	1459 (17.1)	3 (0)	14.45 [13.2, 21.7]
Chronic pulmonary disease *n* (%)	1524 (17.9)	0 (0)	17.5 [17.3, 18.2]
Neuromuscular disease *n* (%)	1064 (12.5)	2 (0)	11.9 [8.7, 16.1]
Existing pacemaker *n* (%)	741 (10.6)	1534 (18)	10.25 [9.5, 10.7]
Dialysis *n* (%)	128 (1.6)	571 (6.7)	1.35 [1.2, 1.9]
NYHA class *n* (%)		2 (0)	
I	176 (2.1)		1.2 [0.8, 1.4]
II	1328 (15.6)		14.15 [10.3, 20.4]
III	5969 (70.0)		75.55 [67.8, 77.1]
IV	1039 (12.2)		10.8 [4.7, 15.7]
NTproBNP median [IQR]	1750.0 [715, 4210]	2375 (27.9)	4063 [3547, 4903]
Creatinine level median [IQR]	90.0 [74, 114]	2 (0)	101.15 [97.3, 105]
eGFR mean (SD)	56.9 (17.6)	0.4	57.3 [55.9, 59.5]
Procedure type *n* (%)		7 (0.1)	
Transapical	491 (5.8)		4.35 [0.2, 12.4]
Transfemoral	7700 (90.4)		90.3 [86.9, 96.4]
Subclavian	203 (2.4)		0.75 [0.2, 3.1]
Direct aortic	123 (1.4)		0.5 [0.2, 2.7]
New permanent pacemaker: yes (%)	720 (10.6)	1731 (20.3)	11 [8.1, 12.1]
Critical procedure: yes (%)	217 (2.5)	2 (0)	2.5 [1.7, 2.7]
Major bleeding: yes (%)	389 (5.5)	1454 (17.0)	3.45 [2.7, 4.1]
New stroke: *n* (%)			
Yes	102 (1.2)	0 (0)	1.15 [1, 1.3]
Yes, ischaemic	49 (0.6)	0 (0)	0.6 [0.4, 1]
ASA: yes (%)	1953 (61.7)	0.1 (0)	58.15 [55.7, 59.1]
NOAC: yes (%)	966 (30.5)	0.1(0)	29.8 [28.9, 31.8]
Warfarin: yes (%)	270 (8.5)	0.1(0)	7.9 [7.4, 9.6]
Trc inhibitor: yes (%)	1310 (41.4)	0 (0)	40.3 [36.2, 43.9]

### Quality indicator attainment

A total of (88.8%) out of the 27 QIs for TAVI were captured from available registry data, 21 (91.3%) directly from existing SWENTRY and SCAAR variables, and 3 (13.0%) after minor modifications. All calculated QIs, except three, were available for analysis throughout the study period. Details regarding attainment are presented in *Table 2*.

**Table 2:** Overall performance and variation characteristics according to the 2023 European Society of Cardiology quality indicators for transcatheter aortic valve implantation

Characteristic	Status	*n* (%)	Missing n (%)	Female	Male	Age ≥ 82	Age < 82
STRUCTURAL QIs							
Numerator: Number of TAVI centres with on-site cardiac surgery. Denominator: Number of TAVI centres	Applies for all centres	8	0				
Numerator: Number of TAVI centres in which regular MDT meetings take place to discuss all patients with severe AS. Denominator: Number of TAVI centres.	Applies for all centres	8	0				
Numerator: Centre performing TAVI which perform ≥ 100 procedures annually. Denominator: Number of TAVI centres.	Captured	6 (75.0)	0 (0)	1 (12.5)	3 (37.5)	2 (25.0)	1 (12.5)
Numerator: Number of TAVI centres which participate in a national TAVI registry. Denominator: Number of TAVI centres.	Applies for all centres	8	0				
PATIENT SELECTION							
Numerator: Number of patients undergoing TAVI who have been discussed in an MDT meeting. Denominator: Number of patients who had a TAVI.	Applies for all centres	8524	0	4023 (47.2)	4501 (52.8)	4599 (54)	3925 (46)
Numerator: Number of patients >80 years of age with severe symptomatic AS who have been treated with TAVI. Denominator: Number of patients >80 years of age with severe symptomatic AS.	Not captured						
Numerator: Number of patients with failed SAVR who are treated with ViV TAVI. Denominator: Number of patients with failed SAVR.	Not captured						
RISK STRATIFICATION							
Numerator: Number of patients undergoing TAVI who have their STS or EuroSCORE II score calculated. Denominator: Number of patients who had a TAVI.	Captured	1105 (13.0)	0 (0)	561 (13.9)	544 (12.1)	597 (13.0)	508 (12.9)
Numerator: Number of patients undergoing TAVI who have their frailty assessed (using a validated tool) prior to the procedure. Denominator: Number of patients who had a TAVI.	Captured*	7521 (88.2)	0 (0)	3543 (88.0)	3978 (88.4)	4000 (87.0)	3521 (89.7)
PROMs							
Numerator: Number of patients undergoing TAVI who have their self- reported health status measured using a validated tool measured. Denominator: Number of patients who had a TAVI.	Not captured						
PRE-PROCEDURAL MEASURES							
Numerator: Number of patients considered for TAVI who had a pre- procedural cardiac-gated CT scan. Denominator: Number of patients considered for TAVI.	Captured	6137 (97.5)	69 (1.1)	2852 (97.9)	3285 (97.2)	3189 (97.9)	2948 (97.1)
PROCEDURAL CONSIDERATIONS							
Numerator: Number of patients who had TAVI via the percutaneous TF route. Denominator: Number of patients who had a TAVI.	Captured	7700 (90.4)	7 (0.1)	3619 (90.0)	4081 (90.8)	4210 (91.6)	3490 (89.0)
Numerator: Number of patients who had TF TAVI without general anaesthesia. Denominator: Number of patients who had TF TAVI.	Captured	6248 (81.2)	1 (0)	2887 (79.8)	3361 (82.4)	3379 (80.3)	2869 (82.2)
POST-PROCEDURAL CARE							
Numerator: Number of patients with AF and no recent PCI (within last 3 months) who are treated with OAC monotherapy post-TAVI. Denominator: Number of patients with AF and no recent PCI (within last 3 months) who had a TAVI.	Captured	742 (75.5)	1 (0.1)	306 (78.7)	436 (73.4)	414 (76.4)	328 (74.4)
Numerator: Number of patients with no indications for OAC or recent PCI (within last 3 months) who are treated with SAPT. Denominator: Number of patients with no indication for OAC or recent PCI (within last 3 months) who had a TAVI.	Captured	661 (37.4)	2 (0.1)	343 (38.0)	318 (36.8)	327 (39.4)	334 (35.6)
OUTCOMES							
Numerator: Number of patients who did not die during the current hospital admission. Denominator: Number of patients who had a TAVI.	Captured	8342 (98.2)	32 (0.4)	3928 (98.0)	4414 (98.4)	4491 (98.0)	3851 (98.5)
Numerator: Number of patients who did not have a stroke during their hospital admission. Denominator: Number of patients who had a TAVI.	Captured	8374 (98.2)	0 (0)	3937 (97.8)	4437 (98.6)	4512 (98.1)	3862 (98.4)
Numerator: Number of patients who did not have a vascular complication during or after the procedure whilst in hospital according to VARC criteria. Denominator: Number of patients who had a TAVI.	Captured	914 (85.3)	7452 (87.4)	480 (84.1)	434 (86.6)	558 (86.6)	356 (83.2)
Numerator: Number of patients who did not have a moderate or severe post deployment aortic regurgitation during the current hospital admission. Denominator: Number of patients who had a TAVI.	Captured	7909 (95.9)	277 (3.2)	3744 (96.5)	4165 (95.3)	4237 (95.5)	3672 (96.4)
Numerator: Number of patients who did not have a reintervention of the aortic valve during the current hospital admission. Denominator: Number of patients who had a TAVI.	Captured	8073 (94.7)	0 (0)	3794 (94.3)	4279 (95.1)	4353 (94.7)	3720 (94.8)
Numerator: Number of patients who did not have open heart surgery during the current hospital admission. Denominator: Number of patients who had a TAVI.	Captured	733 (97.6)	7773 (91.2)	358 (96.8)	375 (98.4)	419 (97.9)	314 (97.2)
Numerator: Number of patients who did not receive a new permanent pacemaker implanted during the current hospital admission. Denominator: Number of patients who had a TAVI.	Captured	6073 (89.4)	1731 (20.3)	2854 (90.1)	3219 (88.8)	3174 (88.4)	2899 (90.5)
Numerator: Number of patients who did not have a coronary obstruction or bail out PCI during current hospital admission. Denominator: Number of patients who had a TAVI.	Captured	736 (98.0)	7773 (91.2)	361 (97.6)	375 (98.4)	421 (98.4)	315 (97.5)
Numerator: Number of patients who did not have a cardiac tamponade during current hospital admission. Denominator: Number of patients who had a TAVI.	Captured	1513 (94.3)	6920 (81.2)	758 (92.4)	755 (96.3)	889 (94.0)	624 (94.8)
Numerator: Number of patients who have undergone a successful TAVI implantation during the current hospital admission. Denominator: Number of patients who had a TAVI.	Captured	8122 (95.4)	8 (0.1)	3837 (95.4)	4285 (95.3)	4352 (94.7)	3770 (96.1)
Numerator: Number of patients who did not have acute kidney injury post-TAVI requiring dialysis during the current hospital admission. Denominator: Number of patients who had a TAVI.	Captured	1047 (97.6)	7451 (87.4)	561 (98.2)	486 (96.8)	635 (98.4)	412 (96.3)
Numerator: Number of patients who did not have a major bleeding during the current hospital admission. Denominator: Number of patients who had a TAVI.	Captured	6680 (94.5)	1454 (17.1)	3085 (93.5)	3595 (95.4)	3537 (94.0)	3143 (0.1)

*Subjective entry in the registry and not based on a validated tool.

For the first domain about structural framework, three QIs: number of TAVI centres with on-site cardiac surgery, centres with regular multidisciplinary team meetings and participation in a national registry applied to all and was not further analysed. Six centres out of eight (75%) reached over 100 annual procedures in 2021. In the second domain about patient selection, two indicators were not possible to calculate from available data and the third QI about multi-disciplinary team (MDT) meetings applied to all patients. In domain three about risk stratification, only 13% had their Society of Thoracic Surgeons (STS) score calculated while the proportion of assessment for frailty was high (88%). Domain four about PROMs was not captured. Domain five about pre-procedural measurements indicated an almost complete attainment to pre-procedural CT scan from its inclusion in the registry in 2016. In domain seven about post-procedural care, a relatively high level of OAC monotherapy was reported in both sexes and in both younger and elder individuals. In patients without indication for OAC, 37.4% were treated with SAPT. For domain eight about outcomes, all outcomes except one had attainment levels at least over 90%. However, for five of the QIs, the level of missing data was high, ranging from between 81–91.2%. In *Table 3*, attainment differences between sexes were reported. Transfemoral route without general anaesthesia and no major bleeding were more common in women. Calculation of STS/European System for Cardiac Operative Risk Model II (EuroSCORE II), stroke, paravalvular regurgitation and cardiac tamponade were less common. Attainment differences between age categories were reported in *Table 4*. Assessment by CT scan, transfemoral route, paravalvular regurgitation, new permanent pacemaker and major bleedings were more common in patients age 82 years or older (above median age). Frailty assessment, transfemoral route without general anaesthesia, successful procedure and new onset dialysis were less common.

**Table 3:** Logistic regression by sex [female vs. male (reference)]

STRUCTURAL Qis		Odds ratio	conf. low	conf. high	*P* value
On-site cardiac surgery	Applies for all				
Regular MDT	Applies for all				
High-performance TAVI centres		1.26	0.946	1.67	0.114
National Registry TAVI centres	Applies for all				
PATIENT SELECTION					
Discussed in an MDT	Applies for all				
Proportion > 80 treated with TAVI	Not captured				
Proportion ViV TAVI of failed SAVR	Not captured				
RISK STRATIFICATION					
STS/EuroSCORE II scoring		0.85	0.75	0.96	0.01
Frailty assessment		1.03	0.91	1.18	0.615
PROMs					
PROM assessment	Not captured				
PRE PROCEDURAL MEASURES					
CT scan		0.739	0.531	1.02	0.069
PROCEDURAL CONSIDERATIONS					
Percutaneous TF route		1.09	0.95	1.26	0.231
TF route without general anaesthesia		1.18	1.05	1.32	0.004
POST-PROCEDURAL CARE					
AF on monotherapy at discharge		0.74	0.55	1.00	0.053
SAPT at discharge		0.94	0.78	1.14	0.555
OUTCOMES					
No in-hospital death		1.28	0.93	1.78	0.13
No in-hospital stroke		1.53	1.11	2.13	0.01
No in-hospital vascular complications		1.23	0.88	1.74	0.233
No in-hospital valve re- intervention		1.17	0.97	1.41	0.108
No in-hospital paravalvular regurgitation		0.73	0.58	0.91	0.006
No open heart surgery		2.1	0.81	6.09	0.142
No new permanent pacemaker implantation		0.89	0.73	1.08	0.246
No coronary obstruction		1.56	0.56	4.7	0.402
No cardiac tamponade		2.14	1.37	3.4	0.001
Successful TAVI		0.97	0.79	1.19	0.762
No new dialysis		0.54	0.24	1.19	0.132
No major bleeding		1.45	1.18	1.78	0

**Table 4:** Logistic regression by median age [age ≥ 82 years vs. < 82 years (reference)]

STRUCTURAL Qis		Odds ratio	conf. low	conf. high	*P* value
On-site cardiac surgery	Applies for all				
Regular MDT	Applies for all				
High-volume TAVI centres		1.09	0.822	1.45	0.545
National Registry TAVI centres	Applies for all				
PATIENT SELECTION					
Discussed in an MDT	Applies for all				
Proportion > 80 treated with TAVI	Not captured				
Proportion ViV TAVI of failed SAVR	Not captured				
RISK STRATIFICATION					
STS/EuroSCORE II scoring		1.01	0.89	1.14	0.932
Frailty assessment		0.77	0.67	0.88	0
PROMs					
PROM assessment	Not captured				
PRE PROCEDURAL MEASURES					
CT scan		1.40	1.02	1.93	0.040
PROCEDURAL CONSIDERATIONS					
Percutaneous TF route		1.35	1.17	1.56	0
TF route without general anaesthesia		0.876	0.780	0.983	0.024
POST-PROCEDURAL CARE					
AF on monotherapy at discharge		1.13	0.84	1.50	0.423
SAPT at discharge		1.17	0.96	1.42	0.112
OUTCOMES					
No in-hospital death		0.76	0.54	1.05	0.097
No in-hospital stroke		0.86	0.62	1.19	0.362
No in-hospital vascular complications		1.31	0.93	1.84	0.121
No in-hospital valve re-intervention		0.97	0.8	1.17	0.767
No in-hospital paravalvular regurgitation		0.8	0.64	0.99	0.043
No open heart surgery		1.34	0.52	3.46	0.542
No new permanent pacemaker implantation		0.8	0.68	0.93	0.005
No coronary obstruction		1.53	0.54	4.41	0.415
No cardiac tamponade		0.85	0.54	1.31	0.464
Successful TAVI		0.72	0.58	0.88	0.002
No new dialysis		2.47	1.12	5.68	0.027
No major bleeding		0.81	0.66	0.99	0.043

### Variation in performance between centres

**Figure 1:**
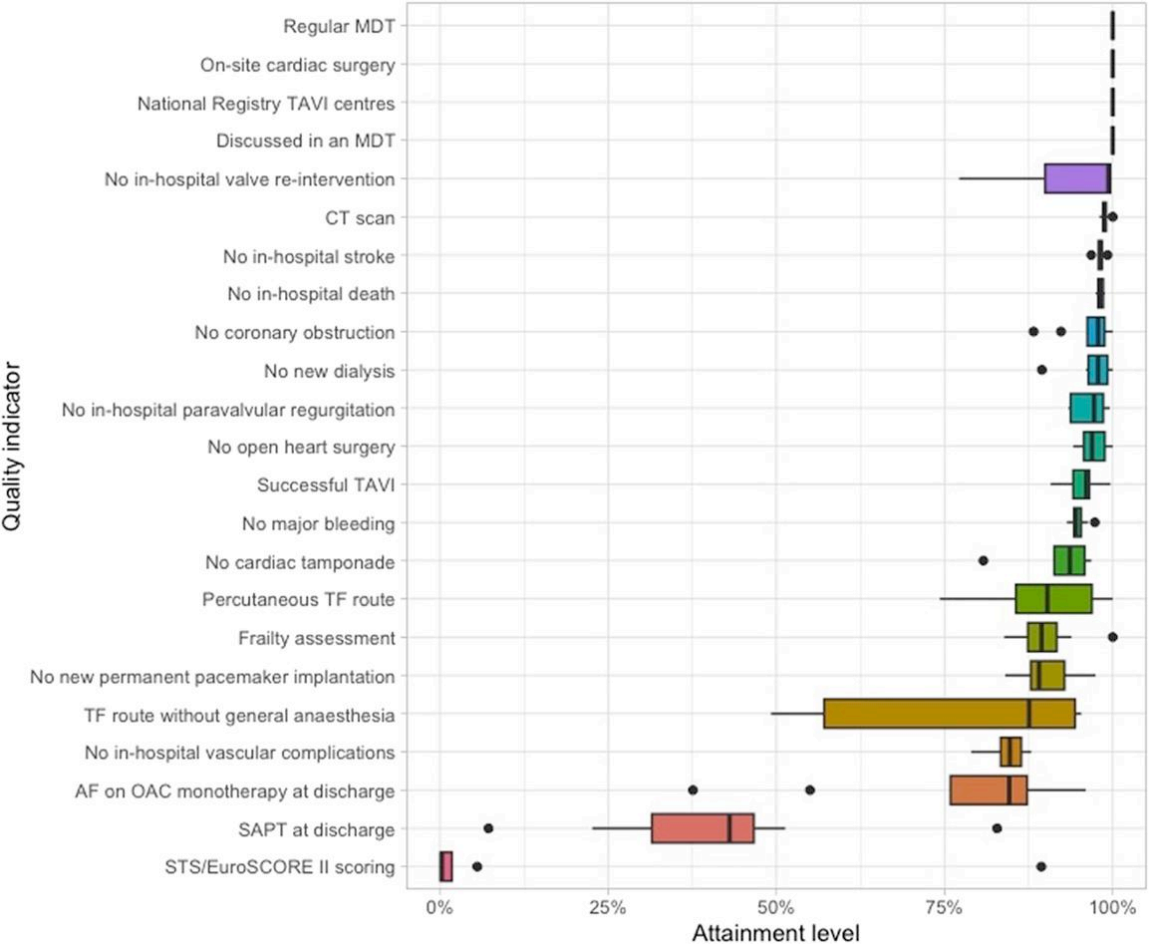
Distribution of centres’ performance according the QIs.

**Figure 2:**
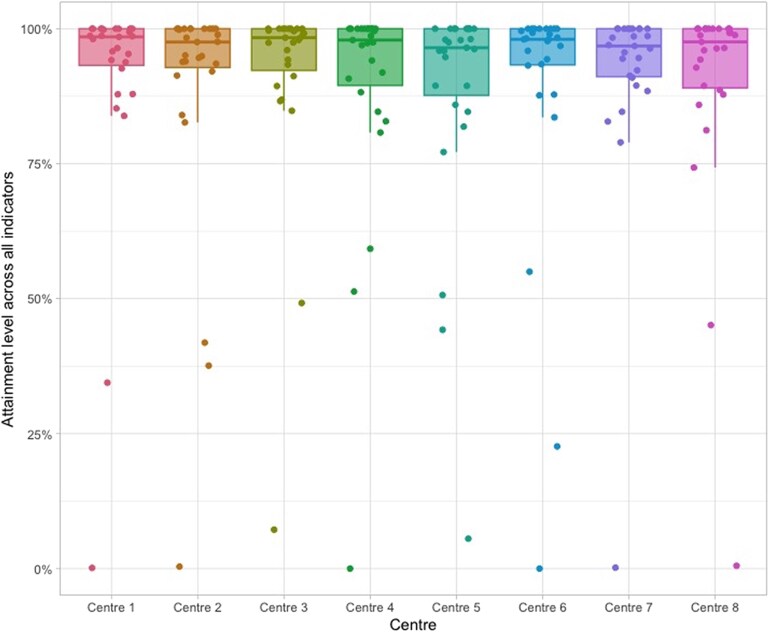
Distribution of total attainment level (all measurable variables) by centre.

Variation of centres’ performance according to the QIs was illustrated in *Figure 1*. The greatest variations were for the QIs concerning choice of approach and anaesthesia with a variation in proportion of transfemoral route of median 90.3%, IQR 85.6–96.9% and a variation in transfemoral route without general anaesthesia with median 87.6%, IQR 57.1–94.4%. During the study period, the preferred procedure type and anaesthetic method changed over time (see Supplementary material online, *Figure S3*), and in a sensitivity analysis consisting of procedures between 2019 and 2021 these variations were substantially smaller, with a variation in proportion of transfemoral route of median 94.2%, IQR 92.5–99.0% and a variation in transfemoral route without general anaesthesia with median 97.2%, IQR 96.3–98.2%. For STS-scoring, the attainment level was low, except for one centre. Two centres also had lower rates of AF on OAC monotherapy at discharge and for SAPT therapy at discharge, while the median attainment was 43%, two centres were outliers at 7.2% and 82.8%, respectively. Total attainment levels by centre were presented in *Figure 2*. Overall, the variation in performance between centres was minor, without clear outliers.

### Association between quality indicators and 1-year all-cause mortality

**Figure 3:**
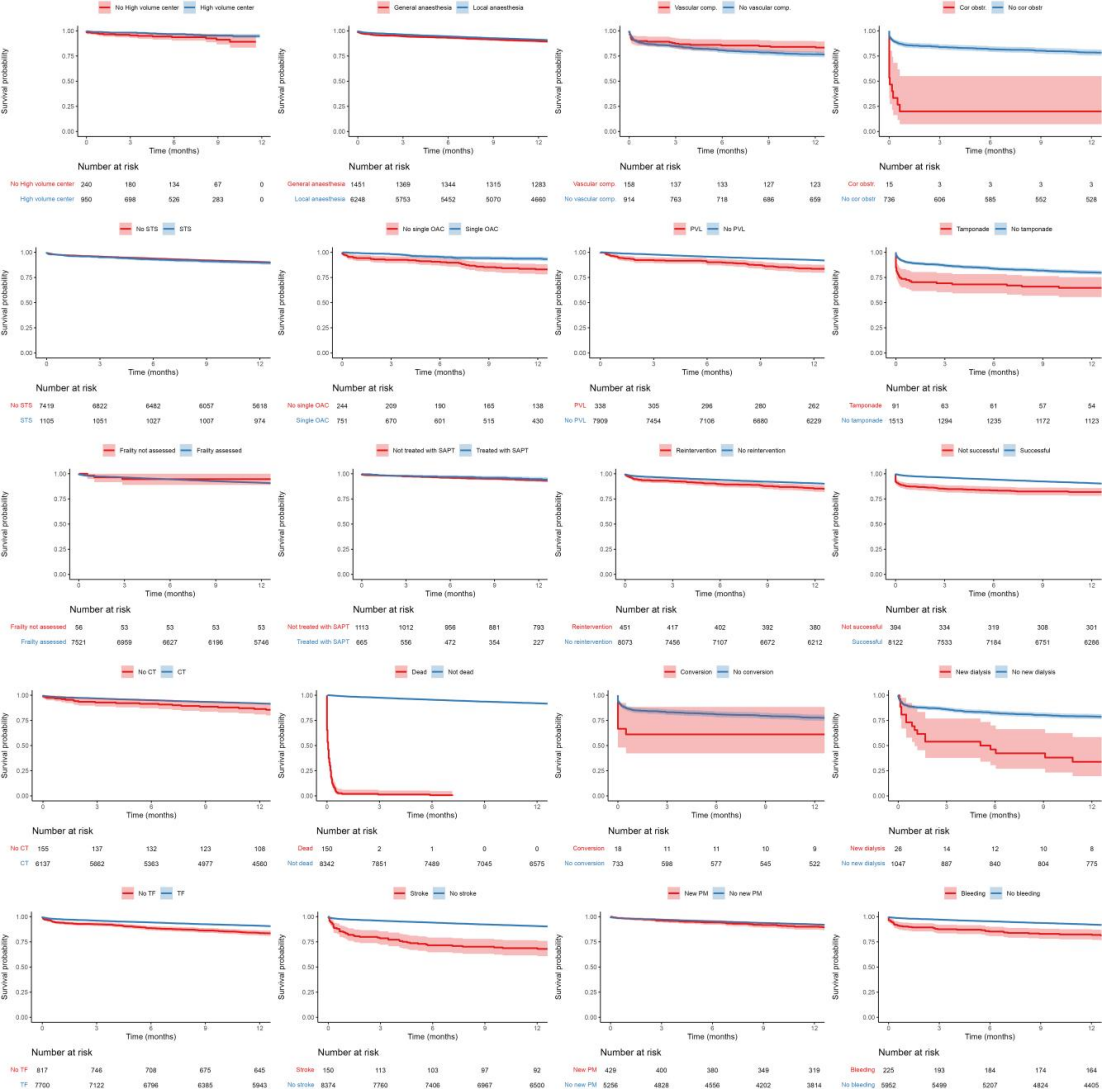
Kaplan-meier curves for all-cause mortality.

**Figure 4:**
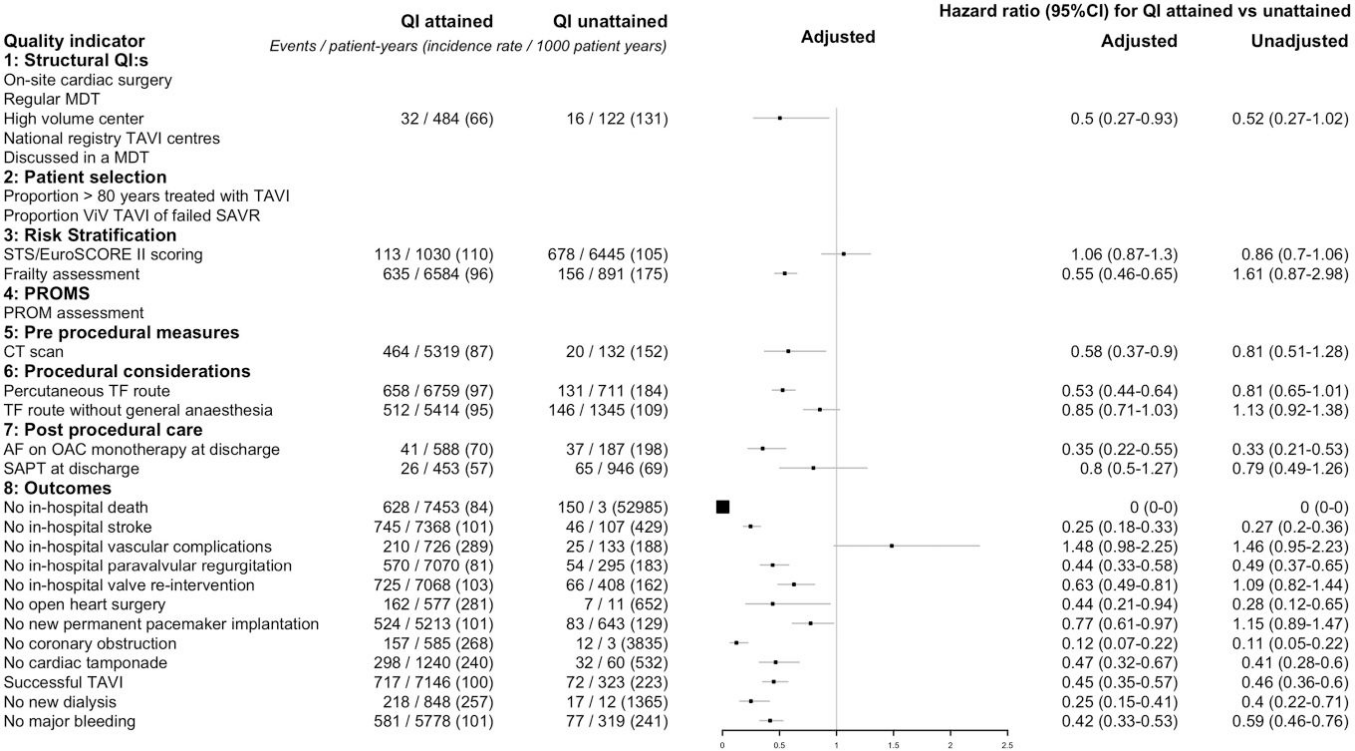
Forest plot of 1-year all-cause mortality.

During the first 12 months, a total of 823 (9.6%) patients died, corresponding to an event rate of 103 events per 1000 patient-years. In *Figure 3*, crude 1-year all-cause mortality for QIs where data was available in this study was illustrated by Kaplan-Meier survival plots. Three QIs, onsite cardiac surgery, regular MDT conferences and participation in a national registry, applied to all patients and therefore could not be assessed. After adjustment, 16 out of 20 measurable QIs (80%) were significantly associated with improved survival at 12 months, see *Figure 4* while 4 QIs, calculation of STS/EuroSCORE II, TF route without general anaesthesia, single antiplatelet therapy at discharge and no in-hospital vascular complications were not associated with improved survival. The QI with the greatest positive impact on improved survival, apart from the QI about in-hospital mortality, was the absence of coronary obstruction (adjusted HR 0.12; 95% CI 0.07–0.22) followed by no new-onset dialysis (adjusted HR 0.25; 95% CI 0.15–0.41) and no in-hospital stroke (adjusted HR 0.25; 95% CI 0.18–0.33). Compared with 1 year, at 30 days, the same indicators were significant except for TF route without general anaesthesia being significant while CT scan and no new pacemaker implantation lost significancy. For long-term follow up (median 2.4 years), TF route without general anaesthesia gained significance while cardiac tamponade lost significancy.

### Association between quality indicators and 1-year cardiovascular mortality

During the first 12 months, 520 cases (6%) of cardiovascular mortality occurred corresponding to an event rate of 70 events per 1000 patient years. Crude 1-year cardiovascular mortality for QIs where data was available in this study was illustrated by Kaplan-Meier survival plots, see Supplementary material online, *Figure S4*. In Supplementary material online, *Figure S5*, adjusted numbers are presented. A total of 17 out of 20 (85%) of the measurable QIs were associated with improved outcome while 4 QIs, calculation of STS/EuroSCORE II, SAPT at discharge and no in-hospital vascular complications were not associated with improved cardiovascular mortality. Apart from the competing event of no in-hospital death, the QIs with the greatest impact on improved survival were no coronary obstruction (HR 0.12; 95 CI 0.06–0.22) followed by no in-hospital stroke (HR 0.18; 95 CI 0.13–0.25) and no new-onset dialysis (HR 0.25; 95 CI 0.15–0.44). Compared with the results at 1 year, at 30 days, CT scan and paravalvular regurgitation lost significancy. For long-term follow up (median 2.4 years), TF route without general anaesthesia gained significance while cardiac tamponade lost significancy.

### Association between quality indicators and 1-year heart failure hospitalization

In total, 775 patients (9.1%) were hospitalized for heart failure within 12 months, corresponding to an event rate of 97 events per 1000 patient-years. In Supplementary material online, *Figure S6*, crude 1-year heart failure hospitalizations for QIs where data was available in this study was illustrated by Kaplan-Meier survival plots. As with 1-year all-cause mortality, three QIs applied to all patients and therefore could not be assessed. Furthermore, for patients who died or experienced coronary obstruction during the in-hospital stay, reliable estimates for QIs measuring these events and heart failure hospitalization could not be calculated, as these patients did not survive beyond discharge and thus could not be rehospitalized. After adjustment, 5 out of 20 measurable QIs (25%; frailty assessment, percutaneous TF route, no in-hospital paravalvular regurgitation, no in-hospital valve reintervention, no new permanent pacemaker implantation) were significantly associated with lower risk of heart failure hospitalization at 12 months, see Supplementary material online, *Figure S7*. The two QIs with the greatest impact were frailty assessment (adjusted HR 0.54; 95% CI 0.45–0.64), and no permanent pacemaker implantation (adjusted HR 0.54; 95% CI 0.4–0.72). The pattern did not differ for long-term follow-up (median 2.4 years) while for 30 days, as compared with the 1-year results, in-hospital valve reintervention was not significant.

## Discussion

In this study, consisting of all patients in all centres undergoing TAVI in Sweden between the years of 2008 to 2021, 89% of the ESC QIs for TAVI could be calculated using available registry data. The majority were derived directly from existing registry variables without modification. Overall, QI attainment was high, with minimal variation between centres. Among the calculated QIs, 80% were associated with increased or decreased risk of all-cause mortality at 12 months. For 1-year cardiovascular mortality, 85% of the measurable QIs were associated to either improved or worse outcome. Furthermore, 25% of the QIs were associated with lower risk of 1-year heart failure hospitalization.

There are several QIs developed by the ESC aiming for different parts of the spectrum of cardiology and there have been earlier studies investigating feasibility and association with clinical outcomes.^17,18^ Similar to this study, those studies reported that most QIs were able to calculate from existing registry data. For the QIs for heart failure, a variation in attainment within and between centres was observed and led to the conclusion that the QIs could be used to measure the quality of care and improve health care outcomes.^18^ In this study, for most indicators, no major differences were observed in attainment levels between centres and attainment levels were high in general. One may then argue that it is more difficult to use the current QIs to compare centres’ performance, as this requires variables that are both associated with outcomes and show differences in attainment level between centres, thus having the potential for improvement. Nonetheless, the majority of the current indicators are associated with mortality. Therefore, if their usage expands to other centres, they could indeed be useful for evaluating clinical practice, as those centres would be expected to achieve similarly high attainment standard.

Women had a lower risk of in-hospital stroke, major bleeding and cardiac tamponade which is in line with previous findings where female sex was associated with favourable outcomes.^19,20^ However, paravalvular regurgitation was more common, where one explanation may be that a small annulus, which is more common in women, is thought to be associated with greater risk of impaired valve haemodynamics.^21,22^ In the regression analysis for outcomes in patients above vs. below median age, as expected, several clinical outcomes (paravalvular regurgitation, new permanent pacemaker bleedings) were more prevalent in patients above median age while a successful procedure was not. However, new-onset dialysis was less common. A plausible explanation is that elderly patients are more carefully selected, before being accepted for valve intervention.

In general, Swedish registries have got high levels of data agreement.^11,23^ However, for some variables in this study, such as serious adverse events during admission, the level of missingness was high. One explanation may be that these variables were not mandatory to complete and thus, they were only filled in when the event actually occurred. Still, when analysing the data, one cannot assume that an empty entry is equivalent to that the event did not occur, although from a clinical perspective, this is probably the case. To avoid such uncertainties, one could consider redesigning the registry form to include either only mandatory fields or pre-filled negative values that must be actively changed if a positive event occurred. However, both these options come with limitations. The former may cause to much effort in filling in the registry which could potentially cause lower levels of data agreement. Furthermore, the latter may lead to an underestimation of missing data. It is also important to make registration easy. The STS score is not automatically calculated in SWENTRY but needs to be done through a separate website which may explain the low attainment levels for STS score found in this study. Moreover, as seen in this study regarding the variations in levels of missingness for new-onset permanent pacemaker and major bleedings the choice of variable construction has a direct effect on data agreement. In summary, one may need to consider different options and choose the one that is assumed to work best and then evaluate and change along the way if needed. For SWENTRY, the current compromise is to mark the most important variables as mandatory.

Some of the proposed QIs apply at the centre level and therefore encompass all patients within a centre, making it difficult to evaluate their association with attainment levels and clinical outcomes. As a result, these QIs may be irrelevant at the centre level, as participating centres automatically fulfil these criteria, which is e.g. the case for centres participating in the EuroHeart TAVI registry.

Most QIs related to outcomes are constructed as negative statements, such as ‘no in-hospital stroke’. However, some indicators were constructed with opposite and positive statements, such as ‘dialysis’. This inconsistency creates unnecessary confusion when interpreting the result and as a consequence, in this study we chose to invert those variables as negative statements to facilitate interpretation. We would therefore recommend future QI Working Groups to have a more consistent approach to the construction of QIs.

### Limitations

Although most QIs were able to capture directly from the SWENTRY registry, not all variables were able to measure and furthermore, there might be underlying considerations not accounted for in the registry for those variables which were able to measure. Ideally, a designated survey would have to be performed to properly account for all aspects of the tested QIs. A strength in the construction of the QIs is that almost all variables have clear definitions. Nonetheless, for some of the variables, there is room for subjective evaluation, which may lead to a discrepancy between what is entered in the registry and what was intended in the definition of the QI. It is also worth noting that the data underlying this study was collected before the publication of the ESCs QI for TAVI in 2024, and if these indicators become widely accepted, it may affect centres’ performances.

## Conclusion

This study reports that most of the ESC 2023 QIs for TAVI could be captured using available Swedish registry data and could be used to evaluate the performance of TAVI care within different domains. The majority of the QIs were associated with 1-year all-cause and cardiovascular mortality and 25% were associated with 1-year new heart failure hospitalization. As such, the QIs may be an important tool for benchmarking and to improve patient outcomes after TAVI.

## Supplementary Material

qcaf146_Supplementary_Data

## Data Availability

The datasets used in this article are legally restricted because of Swedish privacy and secrecy laws and are therefore not publicly available.
