# Supplementary material for: Association between quality of care indicators and clinical outcomes in patients undergoing transcatheter aortic valve implantation: insights from SWEDEHEART
Source: Eur Heart J Qual Care Clin Outcomes. 2025 Dec 3;12(3):366–78. doi: 10.1093/ehjqcco/qcaf146 (PMC13124748; doi:10.1093/ehjqcco/qcaf146)
Supplement: qcaf146_Supplementary_Data [file qcaf146_supplementary_data.docx]

**Supplementary appendix**

**Supplementary Table 1: Baseline characteristics stratified on sex and age category**

| Characteristic | Overall | Female | Male | <82 years | >= 82 years |
| --- | --- | --- | --- | --- | --- |
| n | 8524 | 4024 | 4500 | 3925 | 4599 |
| age median [IQR] | 82 [77, 86] | 83 [78.0, 87.0] | 81 [77, 85] | 77 [73, 79] | 86 [84, 88] |
| sex (male) | 4500 (52.8) | 0 ( 0.0) | 4500 (100.0) | 2303 (58.7) | 2197 (47.8) |
| BMI mean (SD) | 26.9 (8.4) | 26.9 (10.1) | 26.8 (6.4) | 28.2 (10.3) | 25.8 (6.0) |
| Year category n (%) |  |  |  |  |  |
| 2007-2012 | 847 (9.9) | 409 (10.2) | 438 (9.7) | 335 ( 8.5) | 512 (11.1) |
| 2013-2018 | 3921 (46.0) | 1928 (47.9) | 1993 (44.3) | 1684 (42.9) | 2237 (48.6) |
| 2019-2022 | 3756 (44.1) | 1687 (41.9) | 2069 (46.0) | 1906 (48.6) | 1850 (40.2) |
| Hypertension yes (%) | 6484 (76.1) | 3062 (76.1) | 3422 (76.1) | 2967 (75.6) | 3517 (76.5) |
| Diabetes yes (%) | 2152 (25.3) | 868 (21.6) | 1284 (28.5) | 1246 (31.8) | 906 (19.7) |
| Prior myocardial infarction yes (%) | 360 (4.2) | 137 (3.4) | 223 (5.0) | 148 (3.8) | 212 (4.6) |
| Prior PCI yes (%) | 2301 (27.0) | 785 (19.5) | 1516 (33.7) | 1041 (26.5) | 1260 (27.4) |
| Prior stroke n (%) |  |  |  |  |  |
| No | 7481 (87.8) | 3557 (88.4) | 3924 (87.2) | 3432 (87.4) | 4049 (88.0) |
| Yes | 1041 (12.2) | 466 (11.6) | 575 (12.8) | 491 (12.5) | 550 (12.0) |
| Atrial fibrillation n (%) | 3148 (36.9) | 1341 (33.3) | 1807 (40.2) | 1315 (33.5) | 1833 (39.9) |
| Peripheral vessel disease n (%) | 1459 (17.1) | 637 (15.8) | 822 (18.3) | 760 (19.4) | 699 (15.2) |
| Chronic pulmonary disease n(%) | 1524 (17.9) | 726 (18.0) | 798 (17.7) | 911 (23.2) | 613 (13.3) |
| Neuromuscular disease n (%) | 1064 (12.5) | 604 (15.0) | 460 (10.2) | 485 (12.4) | 579 (12.6) |
| Existing pacemaker n(%) |  |  |  |  |  |
| No | 6248 (89.4) | 2989 (91.7) | 3259 (87.3) | 3015 (90.5) | 3233 (88.4) |
| Yes | 741 (10.6) | 269 ( 8.3) | 472 (12.6) | 317 (9.5) | 424 (11.6) |
| Dialysis n (%) | 128 (1.6) | 39 (1.0) | 89 (2.1) | 102 (2.8) | 26 (0.6) |
| NYHA class n (%) |  |  |  |  |  |
| I | 176 (2.1) | 83 (2.1) | 93 (2.1) | 102 (2.6) | 74 (1.6) |
| II | 1328 (15.6) | 605 (15.0) | 723 (16.1) | 686 (17.5) | 642 (14.0) |
| III | 5969 (70.0) | 2874 (71.4) | 3095 ( 68.8) | 2660 (67.8) | 3309 (72.0) |
| IV | 1039 (12.2) | 455 (11.3) | 584 (13.0) | 468 (11.9) | 571 (12.4) |
| NTproBNP median [IQR] | 1750 [715, 4210] | 1710 [705, 3910] | 1780 [725, 4440] | 1312 [540, 3500] | 2110 [939, 4630] |
| Creatinine level median [IQR] | 90 [74, 114] | 80 [67, 100] | 100 [83, 125] | 89 [72, 114] | 92 [75, 114] |
| eGFR mean (SD) | 56.9 (17.6) | 62.4 (16.9) | 52.1 (16.8) | 60.6 (19.7) | 53.8 (15.0) |
| Procedure type n(%) |  |  |  |  |  |
| Transapical | 491 (5.8) | 215 (5.3) | 276 (6.1) | 259 (6.6) | 232 (5.0) |
| Transfemoral | 7700 (90.4) | 3619 (90.0) | 4081 (90.8) | 3490 (89.0) | 4210 (91.6) |
| Subclavian | 203 (2.4) | 107 ( 2.7) | 96 (2.1) | 99 (2.5) | 104 ( 2.3) |
| Direct aortic | 123 (1.4) | 80 (2.0) | 43 (1.0) | 73 (1.9) | 50 (1.1) |
| New permanent pacemaker: no (%) | 5257 (92.5) | 2443 (92.9) | 2814 ( 92.1) | 2559 (92.9) | 2698 (92.1) |
| Critical procedure: yes (%) | 217 ( 2.5) | 80 ( 2.0) | 137 (3.0) | 137 ( 3.5) | 80 ( 1.7) |
| Major bleeding: yes (%) | 226 ( 3.7) | 119 ( 4.2) | 107 (3.2) | 99 ( 3.3) | 127 ( 4.0) |
| New stroke n(%) |  |  |  |  |  |
| Yes | 102 (1.2) | 64 (1.6) | 38 (0.8) | 46 (1.2) | 56 (1.2) |
| Yes, ischemic | 49 (0.6) | 23 (0.6) | 26 (0.6) | 18 (0.5) | 31 (0.7) |
| ASA: yes (%) | 5737 (67.3) | 2705 (67.2) | 3032 (67.4) | 2736 (69.7) | 3001 (65.3) |
| NOAC: yes(%) | 1938 (22.7) | 870 (21.6) | 1068 (23.7) | 853 (21.7) | 1085 (23.6) |
| Warfarin: yes(%) | 1711 (20.1) | 761 (18.9) | 950 (21.1) | 715 (18.2) | 996 (21.7) |
| Trc inhibitor: yes(%) | 4535 (53.2) | 2101 (52.2) | 2434 (54.1) | 2177 (55.5) | 2358 (51.3) |

**Supplementary Figure 1: Number of transcatheter aortic valve procedures over time**

**
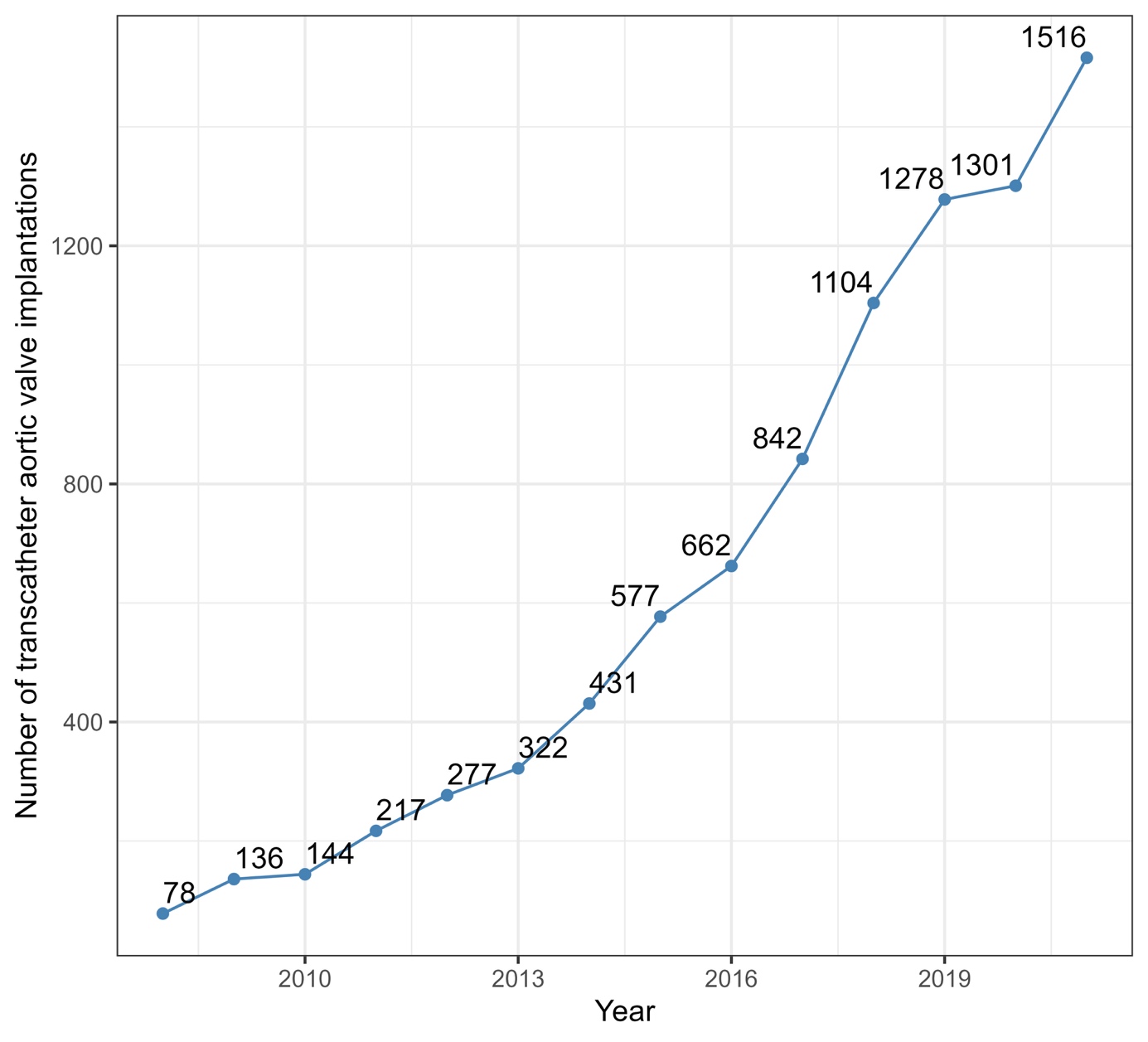
**

**Supplementary Figure 2: Missing frequencies over time for (A) new permanent pacemaker and (B) major bleeding**

**
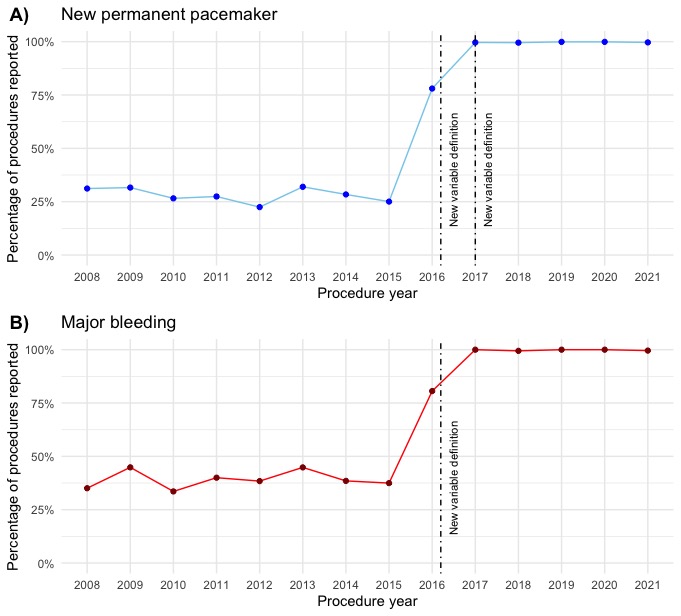
**

**Supplementary Figure 3: Trends in (A) choice of procedural route and (B) in choice of anaesthetic method**

**
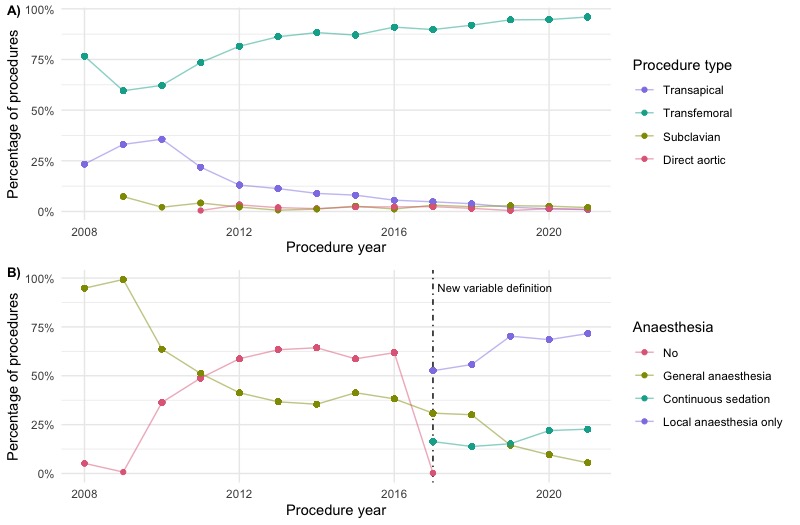
**

**Supplementary Figure 4: Kaplan-meier curves for cardiovascular death**

**
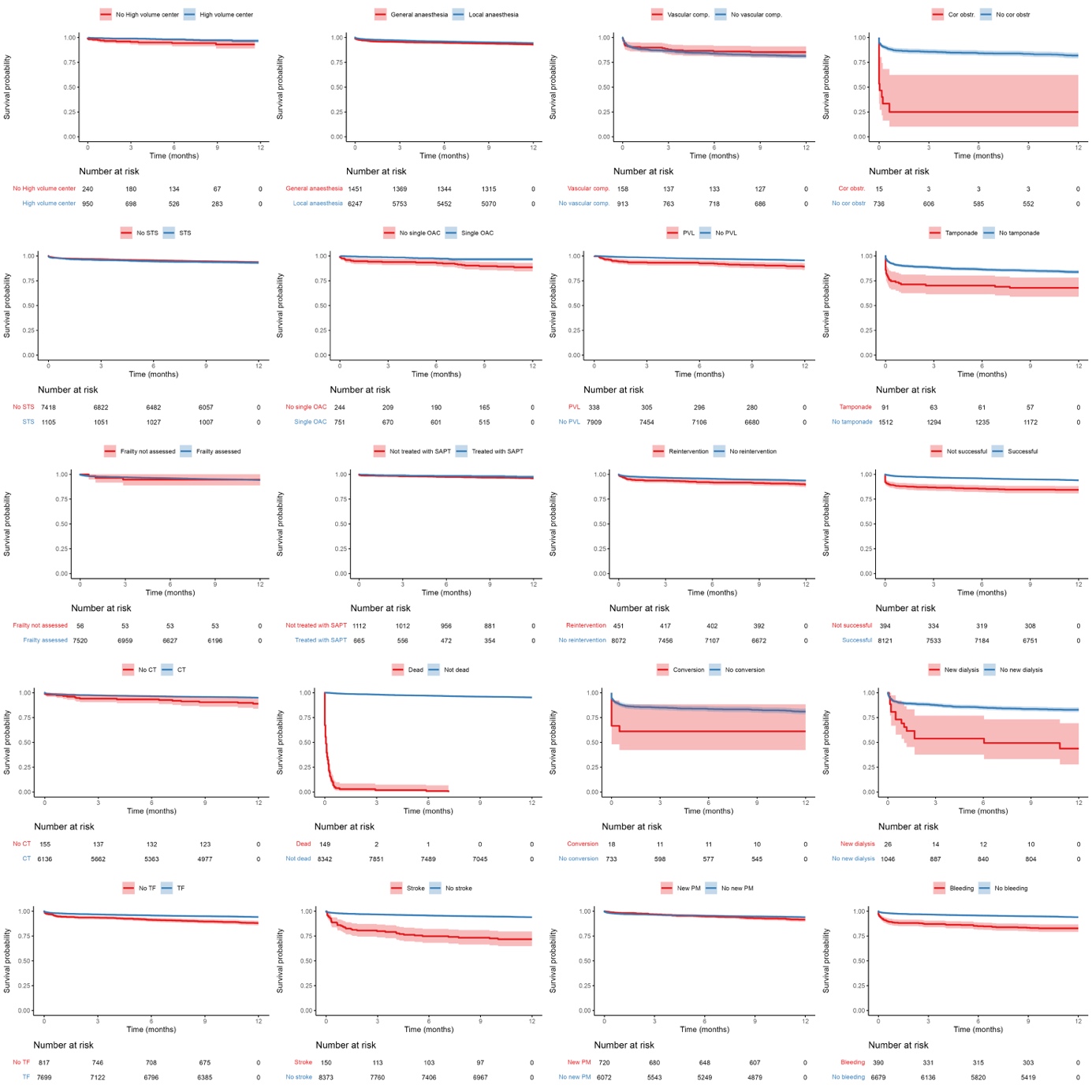
**

**Supplementary Figure 5: Forest plot of 1-year cardiovascular death**

**
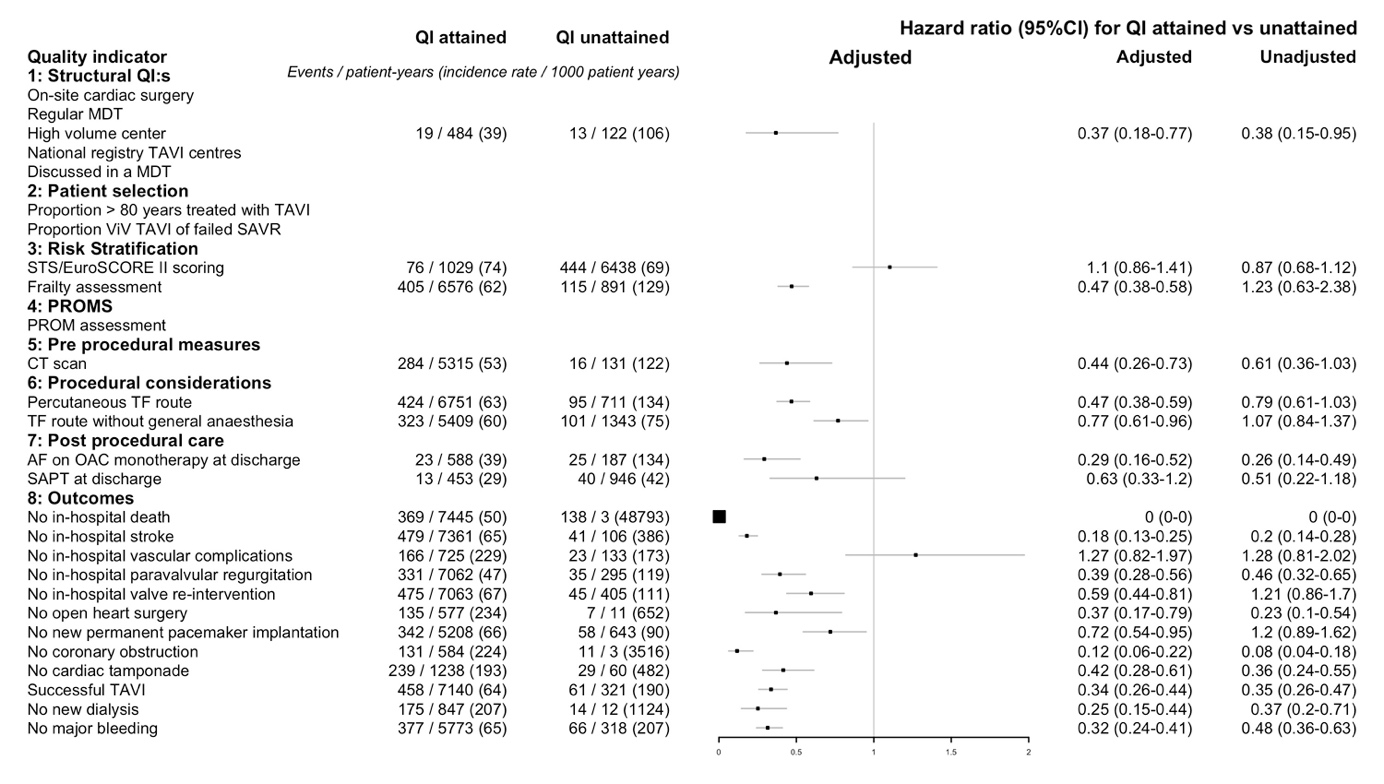
**

**Supplementary Figure 6: Kaplan-meier curves of heart failure hospitalisation**


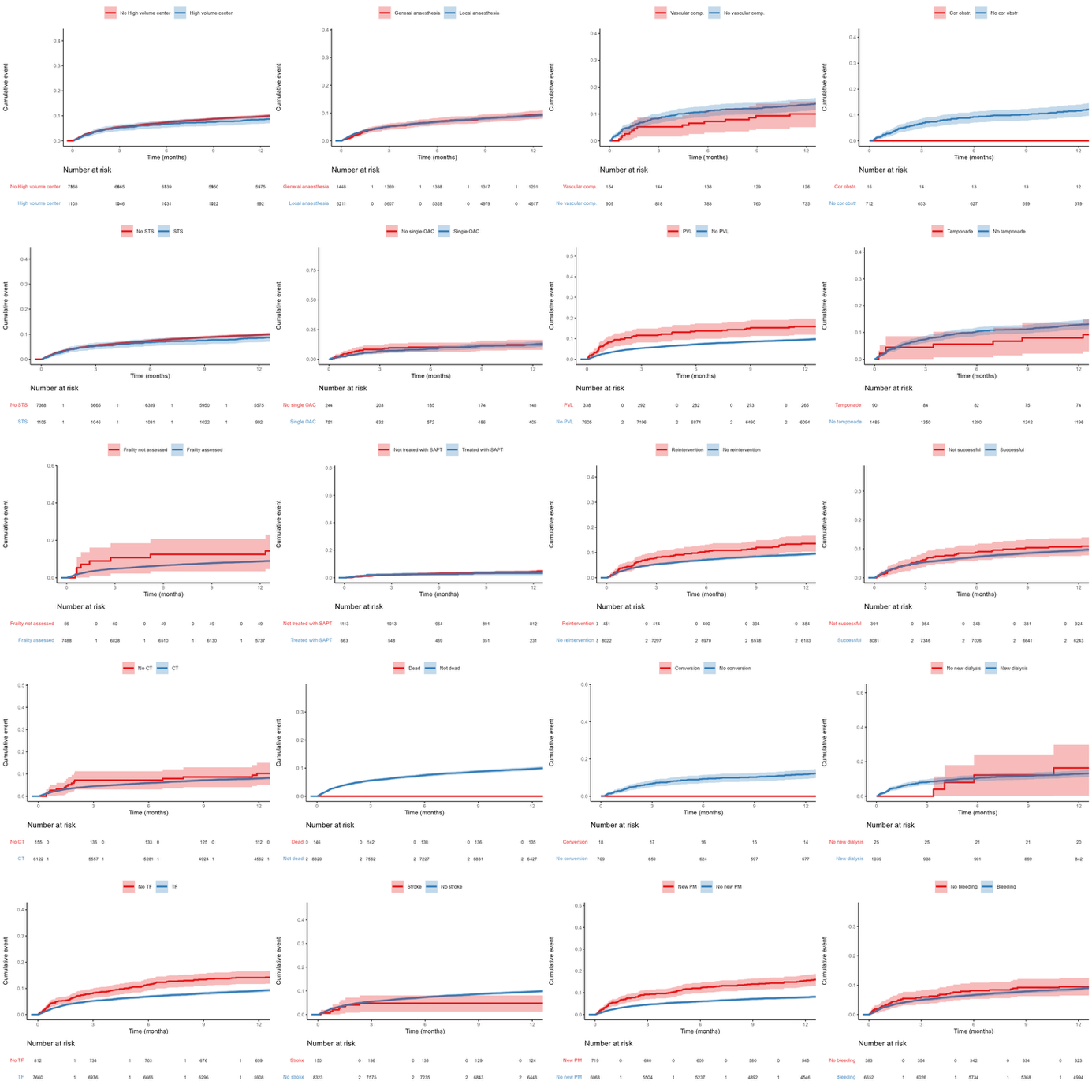


**Supplementary Figure 7: Forest plot of 1-year heart failure hospitalisation**

**
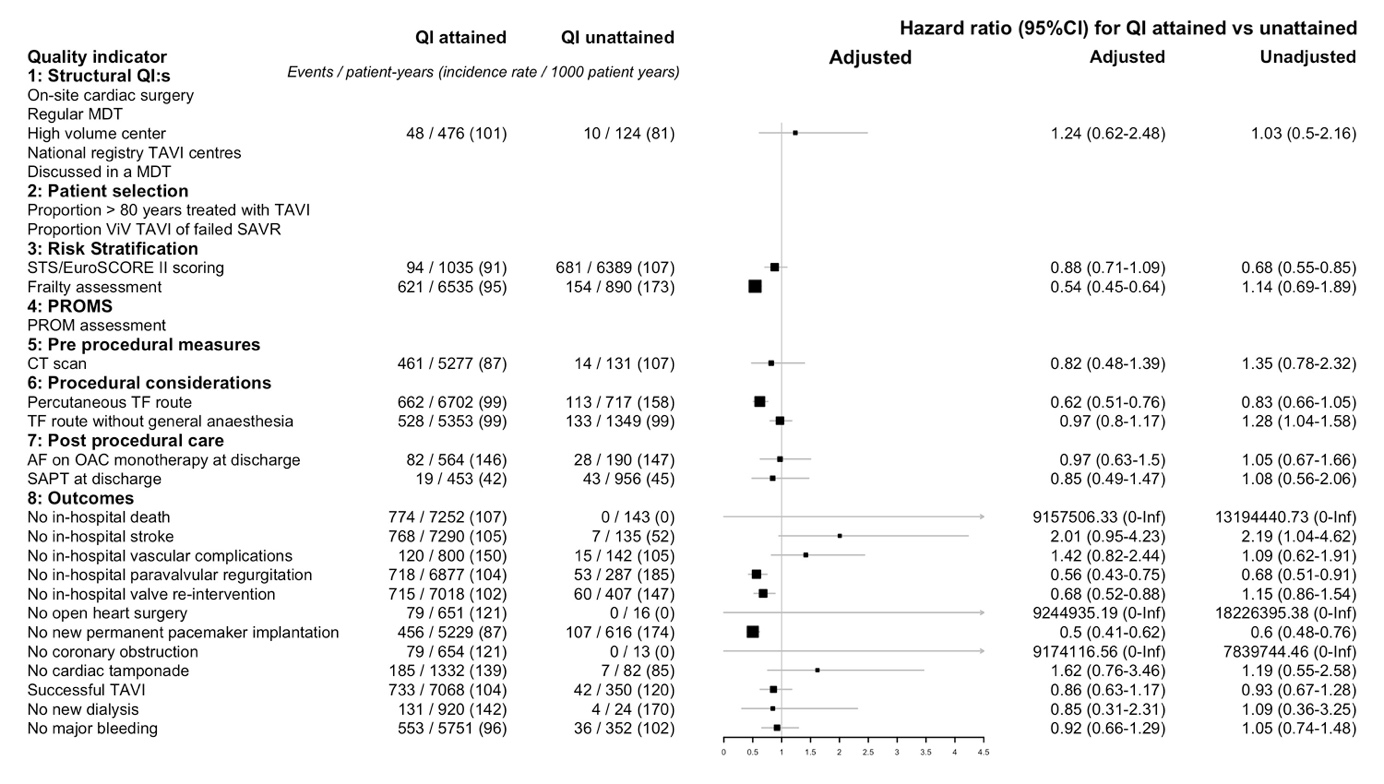
**
